#  Randomised evaluation of pre-notification of trial participants before self-report outcome data collection to improve retention: SWAT86

**DOI:** 10.1177/26320843221098427

**Published:** 2022-05-07

**Authors:** Christopher J Sutton, Sarah Cotterill, Denise Forshaw, Sarah Rhodes, Alexandra Haig, Alison Hammond

**Affiliations:** 1 School of Health Sciences, 5292 The University of Manchester, Manchester, UK; 2 Lancashire Clinical Trials Unit, 6723 University of Central Lancashire, Preston, UK; 3 School of Health Sciences, 7046 University of Salford, Manchester, UK

**Keywords:** study within a trial, data management in clinical trials, alternative trial design and implementation issues, trial methodology, pre-notification, retention, trial conduct

## Abstract

**Background:**

Retention is considered the second highest trial methods priority in the UK after recruitment. There is limited evidence on whether notifying trial participants that a follow-up questionnaire will be sent soon (‘pre-notification’) affects retention.

**Methods:**

This Study Within a Trial (SWAT) evaluated whether sending a pre-notification letter or email around 2 weeks before sending a self-report questionnaire increased retention, in terms of provision of the host trial primary outcome. The SWAT was a randomised, two-arm, parallel-design (1:1 allocation ratio) trial, controlled by ‘no pre-notification letter’. It was embedded within the WORKWELL host trial, which evaluated the impact of job retention vocational rehabilitation on work-related and health-related outcomes of employed people with inflammatory arthritis. The SWAT primary outcome was a valid response for the WORKWELL primary outcome.

**Results:**

Two hundred forty-four trial participants took part in the SWAT. All were included in the analysis. Among those sent a pre-reminder, 100/121 (83%) provided a valid response for the WORKWELL primary outcome, compared to 97/123 (79%) of those not sent a pre-reminder. The estimated adjusted odds ratio was 1.28 (95% confidence interval 0.67–2.42), with a risk difference of 3.8% (95% CI -6.1 to 13.6%), favouring the prereminder. The estimated intervention cost per additional participant retained was £53.42, and the total cost per additional participant retained was £46.52.

**Conclusion:**

Researchers may have a small improvement in trial retention by using pre-notification. The cost per additional participant retained is relatively low. However, further evaluations are merited.

## Introduction

Many trials struggle with participant retention and completion of follow-up questionnaires. A recent UK study found that the median (IQR) retention rate across 151 trials was 89% (79%–97%).^1^ Reminders are generally an effective way of increasing response rates to questionnaires, with some evidence that pre-notification (contacting a participant to say that the trial team will be sending a questionnaire out soon) also provides some benefit, although it is not high certainty evidence.^2^ However, in the trial setting, the recent Cochrane review of methods to improve retention identified only one randomised evaluation of a pre-notification mailing.^3^ In that study,^4^ the effect of a postcard, sent around a month prior to face-to-face collection of the primary outcome was evaluated in a Study Within a Trial (SWAT), nested in the ActWELL trial. Whilst it showed a slight increase in attendance at the data collection appointment (231/274 [84.3%] vs 230/284 [81.0%]: risk difference 3.3%), evidence is currently of ‘low certainty’ because the confidence interval was wide and evidence is based on a single trial. It is therefore important to replicate this intervention to increase the evidence base.

WORKWELL is a pragmatic, multi-centre individually-randomised trial of job-retention vocational rehabilitation for employed people with inflammatory arthritis which, unlike the ActWELL trial, uses participant self-report to collect outcome data.^5^ The MRC-funded PROMETHEUS project resourced a replication of a pre-notification SWAT nested within WORKWELL. Our intervention differed from the ActWELL SWAT in the pre-notification being sent by letter or e-mail rather than postcard because the host trial gave participants a choice of data collection method (postal or online questionnaire). Pre-notification was sent 2 weeks (rather than 4 weeks) in advance of follow-up outcome assessment, to align with the host trial processes and timelines.

The content of all trial communications may impact on questionnaire return rates. A theory-based cover letter intervention (SWAT 24)^6^ was developed using the Theoretical Domains Framework (TDF)^7^ and the Behaviour Change Techniques (BCT) Taxonomy.^8^ Although a theory-based cover letter has now been compared with the use of a standard cover letter in four SWATs, evidence of its effectiveness is still inconclusive, with a non-significant increase in response rate of 3% (95% CI -2 to 8%) and GRADE evidence classed as ‘very low’.^3^ For WORKWELL, the text in the pre-notification communication was informed by the theory and associated text used by Goulao et al.^6^ The WORKWELL pre-notification letter or email was personalised to include the (typed) name of the participant because there is some evidence that personalising may improve response rates in surveys, although this is yet to be supported in a trials context (e.g. in reminders for postal questionnaires^3^).

Whilst many trial process interventions, such as the use of pre-notification, may lead to a marginal gain, there are also cost implications of any such interventions. There has been limited consideration of costs in SWATs. The cost of implementing retention interventions “is an important outcome for evaluators to include in future comparisons so as to provide trial teams with the information they need to make a decision on what will work from an effectiveness and an economic perspective”.^3^ There is little methodological literature on how this should be done, and whether and how any cost savings (through, for example, a reduction in the number of reminders or collection of data via telephone interview following unsuccessful reminders) should be included in the economic evaluation. In the evaluation of costs, we therefore performed both a simple analysis of intervention costs and a more detailed analysis of total costs.

We therefore planned to evaluate the effects of a theoretically-informed, personalised pre-notification letter or email on participants providing a valid response to the primary outcome of the WORKWELL trial, with the aim to add to the body of evidence around the effectiveness and cost-effectiveness of pre-notification on trial retention. We followed the PROMETHEUS SWAT Reporting Guidelines^9^ which are based on the CONSORT 2010 recommendations.^10^

## Methods

### Design

Parallel-group (1:1 allocation ratio) design randomised controlled trial (RCT) embedded within the WORKWELL RCT (the host trial).

### WORKWELL trial PICO

#### Population

People in work, aged at least 18 years, with rheumatoid arthritis, undifferentiated inflammatory arthritis or psoriatic arthritis classified as at medium or high risk of work instability.

#### Intervention

WORKWELL Job retention vocational rehabilitation plus a self-help, written, work-advice pack and usual care.

#### Control

A self-help, written, work-advice pack plus usual care (control intervention);

#### Outcome

The primary outcome is the Work Limitations Questionnaire–25 (WLQ-25) total score.^11^ The WLQ-25 asks about ability to work within the last 2 weeks.

### SWAT setting

WORKWELL Trial participants were recruited from hospital Rheumatology and Therapy departments in England, Wales, and Scotland in the United Kingdom (UK). All patients were seen as outpatients and so the setting for the SWAT was the community, with the expectation that questionnaire responses would be made by WORKWELL trial participants from their own homes.

### Interventions

#### Test intervention

Theoretically-informed, personalised, pre-notification communication in advance of a participant’s 6-month follow-up questionnaire mailing. For participants who elected to complete follow-up questionnaires in hard copy form and return by post, the intervention took the form of a letter sent by post 2 weeks prior to the scheduled mailing of the 6-month questionnaire. For participants who elected to complete follow-up questionnaires online, the intervention was in the form of an email sent 2 weeks prior to the scheduled mailing of an email containing a link to the online questionnaire. The letter and e-mail contained the same form of words. Mailing of a participant’s 6-month questionnaire was scheduled to take place 1 week prior to its due date.

#### Control intervention

No pre-notification communication.

Irrespective of SWAT intervention, participants not responding to the questionnaire mailing within 2 weeks were subject to a standard reminder protocol which involved text, email, reposting and/or telephoning participants, as appropriate.^5^

### Changes to SWAT following commencement

Due to the Covid-19 lockdown during spring/summer 2020, participants who had chosen to complete paper questionnaires were offered the choice of switching to online completion or via telephone interview if they were not able or willing to complete online. This was necessary as it was not possible for the Clinical Trials Unit (CTU) to receive postal questionnaires during this period. This offer occurs subsequent to the sending of the pre-notification to those randomised to that SWAT intervention arm. As a result, the method of delivery of pre-notification was still the participant’s chosen method of questionnaire response. In September 2020, an alternative Freepost PO Box was set up to receive postal questionnaires. Accordingly, not all participants received their follow up questionnaire via their originally chosen method between March and September 2020.

Also due to the Covid-19 pandemic, a number of employed participants were unable to go to work at the 6-month follow-up period for reasons unanticipated when the SWAT was designed (e.g. participants on the UK Government furlough or self-employed income support (SEIS) schemes, or shielding and unable to work from home (and either on furlough, SEIS or receiving statutory sick pay)) and therefore it was not possible for them to complete the WLQ-25 questionnaire. For this reason, there was a deviation from the SWAT protocol in the definition of the primary outcome measure; a response to the 6-month questionnaire that indicates that a person is ‘not working’ (although still in employment) is now included as a valid response for the primary outcome, with the primary outcome listed in the protocol now being reported as a secondary outcome.

### Eligibility criteria

All participants within the WORKWELL trial, except those who had withdrawn or were known to have died prior to the planned mailing of the 6-month outcome questionnaire were eligible.

### Ethics

Participants became part of the SWAT without additional recruitment or consent (over and above consent for WORKWELL), as the SWAT was included within the WORKWELL trial protocol. Ethics approval for this SWAT was obtained from the West Midlands – Solihull Research Ethics Committee (18/WM/0327) as part of the WORKWELL approval process.

### Outcome measures

#### Primary

• Valid response for the WORKWELL trial primary outcome (yes/no) (i.e. usable outcome data for the primary outcome measure (either WLQ-25 total score (5)) obtained by any means, or response that indicates that the participant was not working (for whatever reason) at 6 months.

#### Secondary

• Valid score for the WLQ-25;
• Valid response for WORKWELL trial primary outcome *without reminder* (yes/no)
• Questionnaire returned (yes/no)
• Number of reminders sent (0–3 reminders);
• Time to response [or ceasing follow-up] (days);
• Cost of the intervention (£) per participant retained (where “participant retained” was defined as “one who provided a valid response to the WORKWELL trial primary outcome”);
• Total cost (£) per participant retained.

The costs were estimated as follows, where staff costs used a Research Assistant grade with 2019–2020 academic year Higher Education Single Pay Spine Point 20 (£25,217).

#### Test Intervention costs

For those who chose to receive questionnaires by post, the item costs of printing the pre-notification letter (£0.01), envelopes (£0.04) and postage (£0.65) were included (total £0.70), plus the item staff costs for filling and labelling the envelope (estimated item cost £1.61 [7 min @ £0.23/minute]). For those who chose to receive questionnaires electronically, the item staff cost of sending the pre-notification email was also estimated to be £1.61 (7 min at £0.23 per minute). The costs for preparing the content of the pre-notification letter/email were relatively modest and have not been included: it would be anticipated that this would be largely a one-off research cost and that, should the intervention be applied more widely, very similar text would be used. This resulted in a total item cost of £2.31 for those who opted for a postal questionnaire and £1.61 for those who opted for an electronic questionnaire.

#### Control intervention cost

Zero.

#### Reminder costs

Item staff costs were: Telephone reminder (£0.92 if no answer; £1.15 if a message was left on voicemail; £1.38 if a call was answered); additional time for a call when answered (£1.15); additional time for collection of the minimum data set (£6.44); additional time of collecting a withdrawal data set (£1.15); additional time for collecting item missing data (£4.14); sending a reminder text (£1.84); sending a reminder email (£1.84); re-sending a questionnaire web-link (£1.61); re-sending a postal questionnaire (£1.61); action unknown - only initialled as action (£1.84). Total direct non-staff costs for postal questionnaire mailings were estimated in a similar way to the direct costs for the pre-notification letter (as this was replaced by a cover letter: total £0.70), to which was added the item cost of professional printing of a questionnaire (£1.50). Actual telephone call and text costs were excluded as these were minimal given that the University central service contracts were planned to be used for their delivery.

### Sample size

The SWAT sample size was restricted to a maximum of the size of the host trial, WORKWELL, within which 249 participants were randomised.

### Randomisation

Randomisation was stratified by WORKWELL trial arm and chosen method of 6-month questionnaire receipt (postal vs electronic). It was implemented by Lancashire CTU using the online SealedEnvelope system. When a trial participant reached 3 weeks prior to the date when their 6-month outcome was due for collection, a staff member checked whether the participant had withdrawn or was known to have died. If they were still eligible for WORKWELL trial follow-up a CTU staff member entered them into the SWAT and used the randomisation system to generate their allocation, thus ensuring concealment of allocation until a participant was entered into the SWAT.

### Blinding

WORKWELL trial participants were not blinded to either their host trial arm or SWAT intervention arm, but were unaware that they were participating in a randomised SWAT. The SWAT statistical team remained blind to SWAT group allocation until the SWAT Statistical Analysis Plan had been approved by the SWAT team and published on Figshare (https://doi.org/10.48420/16854517.v1). The final analysis was, however, performed by the statisticians with knowledge of the SWAT group allocations.

### Statistical methods

The main analysis population was all participants randomised as part of the SWAT according to their allocated SWAT treatment group, regardless of any SWAT protocol deviations.

Baseline participant data, and the primary and secondary outcome measures were summarised, using frequency (%), mean (SD) or median (IQR), as appropriate) both overall and by SWAT group allocation.

Comparison of the primary outcome between the pre-notification group and the no pre-notification group used binary logistic regression, including the randomised group factor and adjusting for stratification variables (WORKWELL trial treatment allocation; chosen mode of response). Odds ratios (OR) for the between-groups relative difference in proportions completing the questionnaire were estimated, and presented in conjunction with descriptive statistics of the number and percentage of respondents in each group, and estimates of the risk difference (RD). Both OR and RD are presented as point estimate and 95% confidence interval (CI). Time to response (measured from day first sent) was compared between the groups using Cox regression, adjusted for WORKWELL treatment allocation and chosen mode of response. The date of response was defined by when the response first reached the CTU team. One day was added to all response times to avoid zero survival times. Results are presented as a hazard ratio (HR) and related 95% CI; median time to response in each group is also presented. Kaplan-Meier curves are also presented. Number of reminders by group are summarised as median (IQR) and compared using a Mann-Whitney U test.

For the analysis of the additional cost per additional participant retained we present a crude analysis of the ratio of the average pre-notification intervention cost, divided by the corresponding difference in proportions providing valid response for WORKWELL trial primary outcome. However, as a secondary analysis, we also included the costs of the reminder process, thus performing a fuller cost-effectiveness analysis.

Analysis of the other secondary outcomes was performed using the same method as for the primary outcome.

#### Missing data

Missing data were not expected for the stratification variables, nor any of the outcome variables. If data were missing on the variable actual method of 6-month questionnaire delivery, this analysis was based on available data only.

#### Sensitivity analyses

Sensitivity analyses used were

1. Repetition of primary and secondary outcome analysis with no adjustment for stratification variables.
2. Use of the variable ‘Method of 6-month questionnaire delivery’ instead of ‘Chosen method of 6-month Questionnaire receipt’ for adjustment (primary outcome only).
3. As the WORKWELL Trial indicated that participants would normally only be asked for questionnaire responses for a maximum of 8 weeks (56 days), so the analysis of this outcome was repeated with censoring 63 days after the initial posting of the questionnaires (to allow for the posting being 7 days prior to the due date).

#### Subgroup analyses

Primary outcome and secondary outcomes with subgroups for the chosen response mode, implemented by adding its interaction with SWAT group.

#### Software

All analyses were performed using Stata IC 14.

## Results

### Recruitment and Participant flow

Five of the 249 WORKWELL trial participants withdrew prior to the point at which the pre-notification was to be sent, meaning that 244 SWAT participants were randomised between September 2019 and August 2021 Figure 1. For the SWAT, follow-up was completed by 21 October 2021.

**Figure 1.**
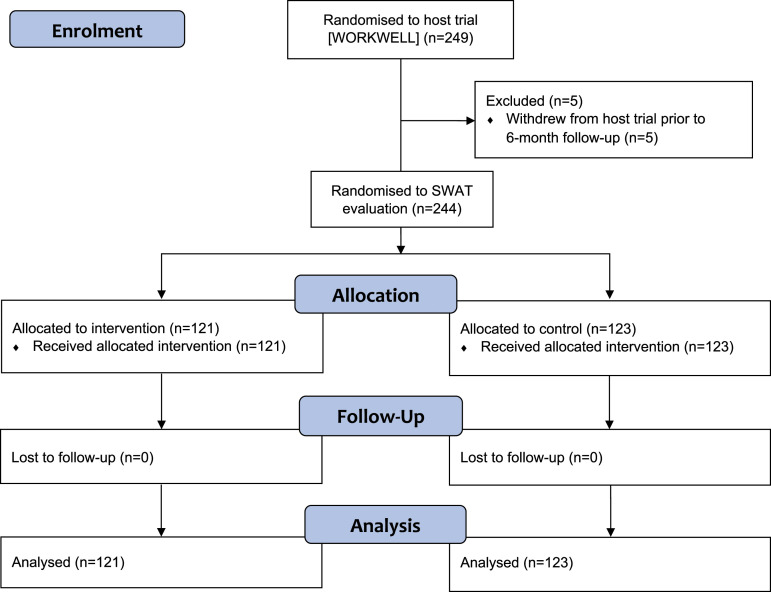
Flow diagram summarizing the flow of participants through the SWAT evaluation.

### Baseline data

Over 80% of SWAT participants were female and 237 (97%) identified as white (Table 1). Mean (SD) age was 48.1 (10.0) years, and 133 (55%) chose to be followed-up by electronic means. All 66 of those in the SWAT intervention arm who had opted for postal questionnaire were sent a postal pre-notification, but 5/55 (9%) of those who had opted for electronic questionnaire were sent a postal pre-notification in error. However, some of those who initially chose to respond by postal questionnaire necessarily were contacted by alternative means due to the COVID-19 pandemic, which resulted in a total of 145 (59%) being sent an email with a link to an online questionnaire, 94 (39%) being sent a postal questionnaire (all of whom had opted to receive the questionnaire via post) and 4 (2%) asking to complete the questionnaire via telephone interview.

**Table 1.** Baseline characteristics by SWAT group allocation and overall.

	Pre-notification *N* = 121	Control *N* = 123	Overall *N* = 244
WORKWELL arm			
A *n* (%)	60 (50)	64 (52)	124 (51)
B *n* (%)	61 (50)	59 (48)	120 (49)
Chosen method of 6-month questionnaire receipt			
Postal n (%)	66 (55)	67 (54)	133 (55)
Electronic *n* (%)	55 (45)	56 (46)	111 (45)
Method of 6-month questionnaire delivery			
Postal *n* (%)	46 (38)	48 (39)	94 (39)
Electronic *n* (%)	72 (60)	73 (59)	145 (59)
Telephone *n* (%)	2 (2)	2 (2)	4 (2)
Missing *n* (%)	1 (1)	0 (0)	1 (<1)
Gender			
Male *n* (%)	19 (16)	26 (21)	45 (18)
Female *n* (%)	102 (84)	95 (77)	197 (81)
Prefer not to say *n* (%)	0 (0)	2 (2)	2 (1)
Ethnic group			
White *n* (%)	118 (98)	119 (97)	237 (97)
Non-white *n* (%)	3 (2)	3 (2)	6 (2)
Prefer not to say *n* (%)	0 (0)	1 (1)	1 (<1)
Age			
Mean (SD)	48.1 (10.0)	49.3 (9.6)	48.7 (9.8)

### Outcomes and estimation

**Figure 2.**
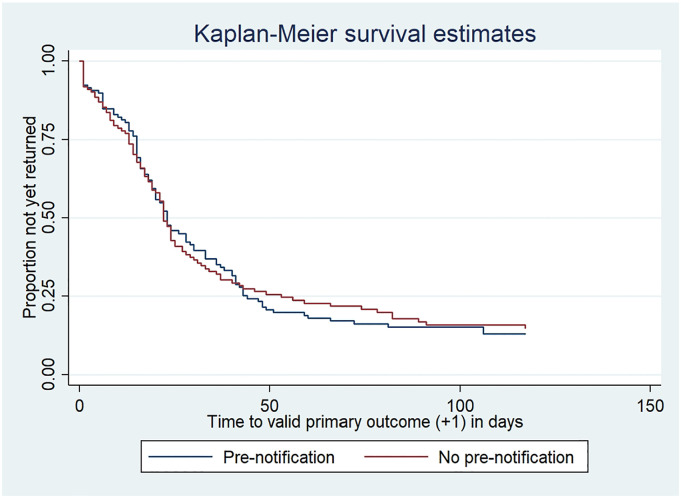
Kaplan-Meier survival estimates of time (+1 day) to participant return of valid primary outcome data.

For the SWAT primary outcome, 100/121 (83%) of the SWAT intervention group and 97/123 (79%) of the control group provided a valid response to the WORKWELL trial primary outcome (OR 1.28; 95% CI 0.67–2.42); this equates to a risk difference of 3.8% (95% CI -6.1 to 13.6%) (Table 2), meaning that an estimated additional 3.8% of valid responses to the primary outcome are obtained by using a pre-notification communication. The OR for collecting a valid WLQ-25 score (i.e. excluding those who were not working) was slightly larger (OR 1.40; 95% CI 0.78–2.50), although there was no indication that the intervention increased the odds of obtaining a valid primary outcome without a reminder (OR 0.96; 95% CI 0.56–1.66). Similar percentages of participants returned 6-month questionnaires (88% in each group) and the median number of reminders sent was one for each group. There was also no evidence of a difference in the chance of the questionnaire being returned at any point during follow-up (HR 1.03; 95% CI 0.78–1.36), with the Kaplan-Meier estimates of the probability of questionnaire return showing similar probabilities for the two groups over time, albeit with most of the very late respondents (>63 days after mailing, i.e. more than 8 weeks after the questionnaire due date) being in the control arm. Figure 2.

**Table 2.** Outcome data by SWAT group allocation.

	Intervention	Control	Relative and absolute effects (95% CI)
	N=121	N=123	
Primary			OR^a^
Valid response for WORKWELL trial primary outcome			1.28 (0.67 to 2.42)
			RD
Yes n (%)	100 (83)	97 (79)	
No n (%)	21 (17)	26 (21)	3.8% (-6.1% to 13.6%)
Valid total 6 month WLQ score			OR^a^
Yes n (%)	94 (78)	88 (72)	1.40 (0.78 to 2.50)
No n (%)	27 (22)	35 (28)	
			RD
			6.3% (-4.6% to 17.2%)
Valid response for WORKWELL trial primary outcome without reminder			OR^a^
			0.96(0.56 to 1.66)
Yes n (%)	37 (31)	39 (32)	RD
No n (%)	84 (69)	84 (68)	
			-0.9% (-12.4% to 0.7%)
Questionnaire returned			OR^a^
			1.06(0.49 to 2.33)
Yes n (%)	107 (88)	108 (88)	
No n( %)	14 (12)	15 (12)	RD
			0.6% (-7.4% to 8.7%)
Number of reminders			Median difference
Median (IQR)	1 (0 to 2)	1 (0 to 3)	0(p=0.74)
Time to valid response for WORKWELL trial (days)			HR^a^
Median	23	22	1.03 (0.78 to 1.36)
Mean SWAT			Mean difference
Intervention cost (£)	2.02	0	2.02
Total cost (£)			Mean difference
Mean (SD)	8.69 (7.19)	6.93 (6.58)	1.76

^a^Adjusted for WORKWELL arm and chosen method of delivery.

The cost of the intervention delivered was an average of £2.02 per person (£2.31 for the 71 sent a postal prompt and £1.61 for the 50 participants sent an electronic prompt), resulting in an estimated cost per additional participant retained of £53.42. A fuller cost-effectiveness analysis, taking into account the costs of the reminders process for those who did not respond to the initial mailing, found a total cost per additional participant retained of £46.53.

### Sensitivity analysis

None of the sensitivity analyses showed important sensitivity of the findings to the alternative assumptions made. However, when adjusting the model for the actual method of questionnaire delivery rather than the chosen method, the odds ratios tended to increase slightly (e.g. for the primary outcome, the OR was 1.36 when adjusted for the actual method, compared to 1.28).

### Subgroup analysis

Subgroup analysis was performed by chosen mode of questionnaire delivery to investigate whether the effect of the SWAT intervention might differ between those who opted for postal compared with those who opted for electronic questionnaire completion. For the primary outcome, there was some evidence that any intervention effect might be smaller for those who opted for postal than for electronic delivery (OR = 0.30; 95% CI 0.08–1.13, *p* = 0.075). Similar results were found for key secondary outcomes (Supplemental Table S1). In the electronic choice subgroup, the intervention cost per additional participant retained was £11.48, and total cost per additional participant retained was £6.54; amongst those who opted for postal delivery the percentage of participants retained was slightly lower in the SWAT intervention arm, so no further analysis of costs was performed.

## Discussion

Sending participants a theoretically-informed pre-notification prior to sending a self-report outcome questionnaire may result in a small increase in trial retention. This finding is consistent with that recently reported SWAT^4^ but this remains GRADE low-certainty evidence as there are no other similar evaluations known in a trial setting.^3^ There is one additional SWAT (SWAT77) registered on the SWAT repository (https://www.qub.ac.uk/sites/TheNorthernIreland NetworkforTrialsMethodologyResearch/SWATSWAR Information/Repositories/SWATStore/), so there may be this or other SWATs ongoing to evaluate a pre-notification email, postcard or letter in a trial setting. As recently reported,^4^ there is broader evidence from other settings that pre-notification of questionnaires may lead to a marginal improvement in response rates, so our findings are also consistent with the wider literature. Our subgroup analysis suggested that the effect of pre-notification may be greater for electronic than postal delivery; this finding merits further research.

The cost of pre-notification is low, and the estimated intervention cost per additional participant retained was around £50. Compared with the costs of recruiting participants, and the potential bias in estimates of effectiveness due to attrition, this cost appears low. Furthermore, the total cost per additional participant retained may be lower than this due to the lessening of subsequent reminders and the costs inherent in this process.

Although not investigated here, delays in participants responding may impact adversely on effectiveness estimation as it is questionable how relevant their outcome is to the scheduled time-point. As can be seen from the Kaplan-Meier plots, despite over 80% of the remaining WORKWELL participants providing valid primary outcome data responses, only around 50% had provided this information by around 2 weeks after its due date (3 weeks after mailing). There was, however, no evidence that the pre-notification led to earlier questionnaire responses.

### Strengths and limitations

We performed a randomised evaluation of a pre-notification letter or email 2 weeks prior to a mailing of a hard-copy questionnaire of, or e-mailing a web-link to, a 6-month outcome questionnaire. This text in the letter (or email) was informed by behaviour-change theory, and was reviewed by the WORKWELL Trial Management Group, including a patient/public member, although it is possible that wider review would have led to improved text. However, as with previous SWATs, our SWAT was performed on a predominantly female and white population of working age. It is unclear how well the results will generalise to more diverse populations. There were also a number of issues caused by COVID: participants were not always able to receive their questionnaires via their original chosen mode, although the pre-notifications were mostly sent using the chosen mode of questionnaire delivery. Costs may not generalise to other trials. However, our SWAT has the strength that, unlike most previous SWATs, in addition to a simple consideration of intervention costs, we performed a full cost-effectiveness analysis. In this, we included estimated reminder costs which will be reduced if the intervention is successful in initiating an early response, in addition to the intervention costs. Similarly, our estimate of the effect of a pre-reminder may not generalise to other trials, as the effect may vary according to the population or control group retention rate. However, our control group retention rate is relatively high (79%) so there would be more potential for a greater absolute effect (risk difference) of the intervention on retention.

### Interpretation

Pre-notification may lead to a small increase in response rates to outcome questionnaires. The cost of pre-notification relative to potential gains is low. However, further evaluations of pre-notifications, whether by letter or digital means, and using theoretically-informed text believed likely to maximise any impact, are needed.

### Data sharing

Data will be supplied to the University of York as part of the data sharing agreement for the PROMETHEUS project and will be available from the lead author following publication of the WORKWELL trial.

### Registration

This SWAT is registered as SWAT86 on the SWAT Repository at https://www.qub.ac.uk/sites/TheNorthernIreland NetworkforTrialsMethodologyResearch/SWATSWAR Information/Repositories/SWATStore/

## Supplemental Material

Supplemental Material - Study Within A Trial (SWAT86): Randomised evaluation of pre-notification of trial participants before self-report outcome data collection to improve retention Supplemental Material for Study Within A Trial (SWAT86): Randomised evaluation of pre-notification of trial participants before self-report outcome data collection to improve retention by Christopher J Sutton, Sarah Cotterill, Denise Forshaw, Sarah Rhodes, Alexandra Haig and Alison Hammond in Research Methods in Medicine & Health Sciences
