# Supplementary material for: Randomised evaluation of pre-notification of trial participants before self-report outcome data collection to improve retention: SWAT86
Source: Res Methods Med Health Sci. 2022 May 7;3(4):107–15. doi: 10.1177/26320843221098427 (PMC13021075; doi:10.1177/26320843221098427)
Supplement: Supplemental Material - Study Within A Trial (SWAT86): Randomised evaluation of pre-notification of trial participants before self-report outcome data collection to improve retention [file sj-pdf-1-rmm-10.1177_26320843221098427.pdf]

Table S1: Outcome data by SWAT group allocation, by chosen mode of delivery

	Intervention	Control	Interaction (subgroup comparison)
PRIMARY<br><br>**Valid response for WORKWELL trial primary outcome n(%)**<br><br>Postal<br>Electronic	 <br><br><br><br>53(80)<br>47(85)	 <br><br><br><br>57(85)<br>40(71)	Odds ratio*<br><br><br><br><br>0.30 (0.08 to 1.13)<br>(P = 0.075)
**Valid total 6 month WLQ score n(%)**<br>Postal<br>Electronic	 48(74)<br>45(81)	 51(76)<br>37(66)	Odds ratio*<br>0.39(0.12 20 1.27)<br>(P=0.118)
**Valid response for WORKWELL trial primary outcome without reminder n(%)**<br>Postal<br>Electronic	 20(30)<br>17(31)	 23(34)<br>16(29)	Odds ratio*<br>0.74(0.25 to 2.22)<br>(P=0.591)
**Number of reminders Median(IQR)**<br>Postal<br>Electronic	 1(0 to 2)<br>1(0 to 3)	 1(0 to 2)<br>1(0 to 3)	 N/A
**Time to valid response for WORKWELL trial (days) Median**<br>Postal<br>Electronic	 22<br>23	 22<br>24	Hazard ratio*<br>0.70(0.39 to 1.87)<br>(P=0.211)
**Total Cost (£)**<br>Mean (SD)<br>Postal<br>Electronic	 <br>8.22(6.32)<br>9.24(8.13)	 <br>5.78(5.16)<br>8.32(7.77)	 <br>N/A

*Adjusted for WORKWELL arm. Odds ratios/hazard ratios represent the ratio of the effect for those who opted for postal questionnaire delivery compared to that for those who opted for electronic delivery.
